# Provincial heterogeneity in the management of care cascade for hypertension, diabetes, and dyslipidaemia in China: Analysis of nationally representative population-based survey

**DOI:** 10.3389/fcvm.2022.923249

**Published:** 2022-08-23

**Authors:** Yang Zhao, Kanya Anindya, Rifat Atun, Tiara Marthias, Chunlei Han, Barbara McPake, Nadila Duolikun, Emily Hulse, Xinyue Fang, Yimin Ding, Brian Oldenburg, John Tayu Lee

**Affiliations:** ^1^The George Institute for Global Health, Beijing, China; ^2^The George Institute for Global Health, University of New South Wales, Sydney, NSW, Australia; ^3^The Nossal Institute for Global Health, The University of Melbourne, Melbourne, VIC, Australia; ^4^Department of Global Health and Population, Harvard T.H. Chan School of Public Health, Harvard University, Boston, MA, United States; ^5^Department of Global Health and Social Medicine, Harvard Medical School, Harvard University, Boston, MA, United States; ^6^Department of Public Health, Faculty of Medicine, Public Health and Nursing, Universitas Gadjah Mada, Yogyakarta, Indonesia; ^7^College of Public Health and Management, Binzhou Medical University, Yantai, China; ^8^Chinese Center for Disease Control and Prevention, Beijing, China; ^9^School of Software, Tongji University, Shanghai, China; ^10^Baker Heart and Diabetes Institute, Melbourne, VIC, Australia; ^11^Public Health Policy Evaluation Unit, Department of Primary Care and Public Health, School of Public Health, Imperial College London, London, United Kingdom; ^12^Department of Health Service Research and Policy, Australian National University, Canberra, NSW, Australia

**Keywords:** cardiovascular disease, behavior risk, care cascade, regional disparity, China

## Abstract

**Background:**

This study aims to examine (1) province-level variations in the levels of cardiovascular disease (CVD) risk and behavioral risk for CVDs, (2) province-level variations in the management of cascade of care for hypertension, diabetes, and dyslipidaemia, and (3) the association of province-level economic development and individual factors with the quality of care for hypertension, diabetes, and dyslipidaemia.

**Methods:**

We used nationally representative data from the China Health and Retirement Longitudinal Study in 2015, which included 12,597 participants aged 45 years. Using a care cascade framework, we examined the quality of care provided to patients with three prevalent NCDs: hypertension, diabetes, and dyslipidaemia. The proportion of WHO CVD risk based on the World Health Organization CVD risk prediction charts, Cardiovascular Risk Score (CRS) and Behavior Risk Score (BRS) were calculated. We performed multivariable logistic regression models to determine the individual-level drivers of NCD risk variables and outcomes. To examine socio-demographic relationships with CVD risk, linear regression models were applied.

**Results:**

In total, the average CRS was 4.98 (95% CI: 4.92, 5.05), while the average BRS was 3.10 (95% confidence interval: 3.04, 3.15). The weighted mean CRS (BRS) in Fujian province ranged from 4.36 to 5.72 (*P* < 0.05). Most of the provinces had a greater rate of hypertension than diabetes and dyslipidaemia awareness and treatment. Northern provinces had a higher rate of awareness and treatment of all three diseases. Similar patterns of regional disparity were seen in diabetes and dyslipidaemia care cascades. There was no evidence of a better care cascade for CVDs in patients who reside in more economically advanced provinces.

**Conclusion:**

Our research found significant provincial heterogeneity in the CVD risk scores and the management of the cascade of care for hypertension, diabetes, and dyslipidaemia for persons aged 45 years or more. To improve the management of cascade of care and to eliminate regional and disparities in CVD care and risk factors in China, local and population-based focused interventions are necessary.

## Introduction

China, with a population of 1.3 billion people, has experienced a huge epidemiological change in the last decade, with the health burden moving from infectious diseases to non-communicable diseases (NCDs), including cancer, cardiovascular disease (CVD), diabetes, and respiratory diseases (1). However, the speed and nature of this epidemiological transition varied substantially among provinces, with higher economic development provinces having a larger burden of NCDs (2–5) and provinces with lower higher economic development having a higher burden of communicable, maternal, neonatal, and nutritional disorders (6–9). This variation in epidemiological profile, combined with unequal distribution of healthcare resources among provinces, has major consequences for subnational health policy decisions.

Improved health outcomes and reduced health inequalities for CVDs require accessible high-quality care, which includes early detection, proper treatment, and ongoing monitoring (10). The cascade of care framework is a useful tool for understanding provincial variation in quality of care in managing CVDs. Several cross-sectional studies to date have shown major variation and inadequacies in the management of care cascade for NCDs in low-income and middle-income countries (11–13). However, there are very few published national-representative studies that have examined variation in the management of CVD at subnational level (14). Analyses using care cascade framework enables the estimation of percentages of patients with a particular CVD who are screened, diagnosed, aware of their condition, taking medication, and in control (15, 16). While many studies have been done to examine CVD risk factors and the quality of CVD care in China, most of them have focused on the national or regional level (17–23). Data on the quality of care for CVDs at the provincial level is currently scarce (18).

Furthermore, the relationship between provincial-level economic development, quality of care, and socioeconomic inequalities in quality of care is hardly known in China (18). This study employed data from a nationally representative survey to examine: (1) province-level variations in CVD risk scores and behavioral risk scores for CVDs in China; (2) province-level variations in the proportion of adults with hypertension, diabetes, and dyslipidaemia who have reached each step of the care cascade (awareness of diagnosis, sought treatment, and blood pressure/glycaemic/blood lipids, control); and (3) the association of province-level economic development and individual factors with the management of cascade of care for hypertension, diabetes, and dyslipidaemia.

## Methods

### Data sources

We analyzed the 2015 China Health and Retirement Longitudinal Study (CHARLS) dataset, which is a nationally representative survey of respondents aged 45 and older that collects high-quality data. The CHARLS was done utilizing a multistage stratified probability-proportionate-to-size sampling strategy, with biannual follow-up surveys. The initial sample size was 17,708 people. The CHARLS' objectives, design, and methodology are described in full elsewhere (24). We identified 13,354 respondents with blood test and biomarker information for this investigation. After excluding respondents with missing values (5.7%) for either the dependent or independent variables, the final sample size is 12,597 respondents (the flowchart for subject selection is included in Supplementary Figure S1).

### Cardiovascular health and CVD Risk

To compare the proportion of the population at various levels of CVD risk, we used the recently updated non-laboratory-based WHO CVD risk prediction charts, which are recommended in the WHO Package of Essential Noncommunicable Disease Interventions and Global Hearts Initiative guidelines. They calculate the 10-year risk of a CVD event defined as myocardial infarction or stroke for each sex (25). These equations, developed by the WHO CVD Risk Chart Working Group, are calibrated to 21 areas using CVD incidence data from the Global Burden of Disease research. They incorporate age, smoking status, systolic blood pressure (SBP), and body mass index (BMI). We used the CHARLS data to apply the WHO risk models for East Asia.

Meanwhile, the Cardiovascular Risk Score (CRS) were also calculated for each participant in this study using an eight-component approach, which included smoking, alcohol use, physical activity, sleep, BMI, total cholesterol, blood pressure, and fasting glucose (18, 26). The Behavior Risk Score (BRS) was calculated using data on smoking, alcohol consumption, physical activity, sleep, and BMI. Except for sleep (poor = 1 point; ideal = 0 points), each measure was divided into three levels (poor = 2 points; intermediate = 1 point; ideal = 0 points). Supplementary Table S2 has detailed definitions of “poor,” “intermediate,” and “excellent” values for all metrics. The CRS ranged from 0 to 15, whereas the BRS score was between 0 and 9. The term “perfect cardiovascular health” refers to persons without a history of cardiovascular illness who possess all eight of the ideal cardiovascular health components.

### Management of care cascade for hypertension, diabetes, and dyslipidaemia

Physical examination or medication was used to determine the prevalence of CVDs. The CVDs considered in this study were hypertension, diabetes, and dyslipidaemia, which were quantified using biomarkers or blood tests. In the CHARLS, a trained nurse assessed respondents' SBP and diastolic blood pressures (DBP) three times using the HEM-7112 electronic monitor. SBP ≥140 mmHg and/or DBP ≥90 mmHg were used to diagnose hypertension, as well as the use of hypertension medication (27, 28). Diabetes was defined as having one or more of the following: (1) fasting blood plasma glucose ≥126 mg/dl; (2) HbA1c concentration ≥6.5%; and (3) insulin treatment and/or medication for hyperglycemia (29, 30). Dyslipidaemia was defined as the presence of the following: (1) total cholesterol ≥240 mg/dl and/or; (2) low-density lipoprotein cholesterol ≥160 mg/dl and/or; (3) high-density lipoprotein cholesterol <40 mg/dl and/or; (4) triglyceride ≥200 mg/dl and/or; and (5) use of antidyslipidaemia medication (31, 32).

We used a care cascade framework to examine management of hypertension, diabetes, and dyslipidaemia, which covers in the survey diagnosis, awareness, treatment, and control steps in the management of the conditions. Self-reporting a medical professional's diagnosis of an NCD was characterized as diagnosis or awareness. Respondents with an NCD who were taking medication were classified as being in treatment. Responders who had received treatment for an NCD and were no longer exhibiting symptoms on physical examination were referred to as “controlled.” (See a detailed description in Supplementary Table S5).

### Provincial economic development level

A total of 28 provinces were identified and rated in terms of economic development based on their 2015 Gross Domestic Product (GDP) per capita. To obtain robust estimates for each outcome at the subnational level, we removed seven provinces (Qinghai, Guizhou, Xinjiang, Chongqing, Tianjin, Beijing and Shanghai) with small sample size (e.g. fewer than 200 individuals) from provincial analysis.

### Covariates

This study also included the following covariates in the regression analyses: age, gender, marital status (married and partnered, unmarried and others), education attainment (illiterate, primary school, secondary school, college and above), place of residency (rural, urban), region of economic development (quartile from lowest to highest), household economic status quartiles (annual per capita household consumption expenditure), and social health insurance coverage (yes, no).

### Statistical analysis

At the national level and by province and sociodemographic group, we describe the prevalence of chronic disease risk factors, cardiovascular disease health behaviors, and the prevalence and cascade of care for hypertension, diabetes, and dyslipidaemia. The age-sex-standardized prevalence rates for outcome variables were summarized using the 2010 census population (33).

We used multivariate logistic regression to determine the individual-level drivers of CVD risk variables and outcomes. To examine the associations between economic development and outcomes (such as CVD health and the care cascade for each of the three conditions) at the province level, we plotted economic development against risk factors or cascade indicators for provinces and used Pearson's correlation test to examine the association. Additionally, we used multivariable logistic regressions to examine the connection between specific sociodemographic variables and indicators of the care cascade. To examine sociodemographic relationships with CVD and behavior risk, linear regression models were applied using the logarithm of the risk as the dependent variable. We examined multicollinearity for covariates that were included in our analysis.

The variance inflation factors for multicollinearity diagnostics were all less than five, indicating that the assumption of reasonable independence between predictor variables was met. Sample weights were employed to accommodate for the CHARLS survey's complicated, multistage design. The analysis was conducted using the statistical programme Stata 16.0 (Stata Corp., College Station, Texas).

## Results

### Sample characteristics

Supplementary Table S1 showed the basic characteristics of total 12,597 middle-aged and older participants in China. The mean age of respondents was 60.5 ± 9.7 years, with more than 90% of participants aged 45–74 years old. Females made up half of the participants. 86.5% of persons were married or living unmarried as a couple, while 65.9% were illiterate. Almost half of the middle-aged and older Chinese adults resided in urban areas.

### CVD risk at national and provincial levels

**Figure 1:**
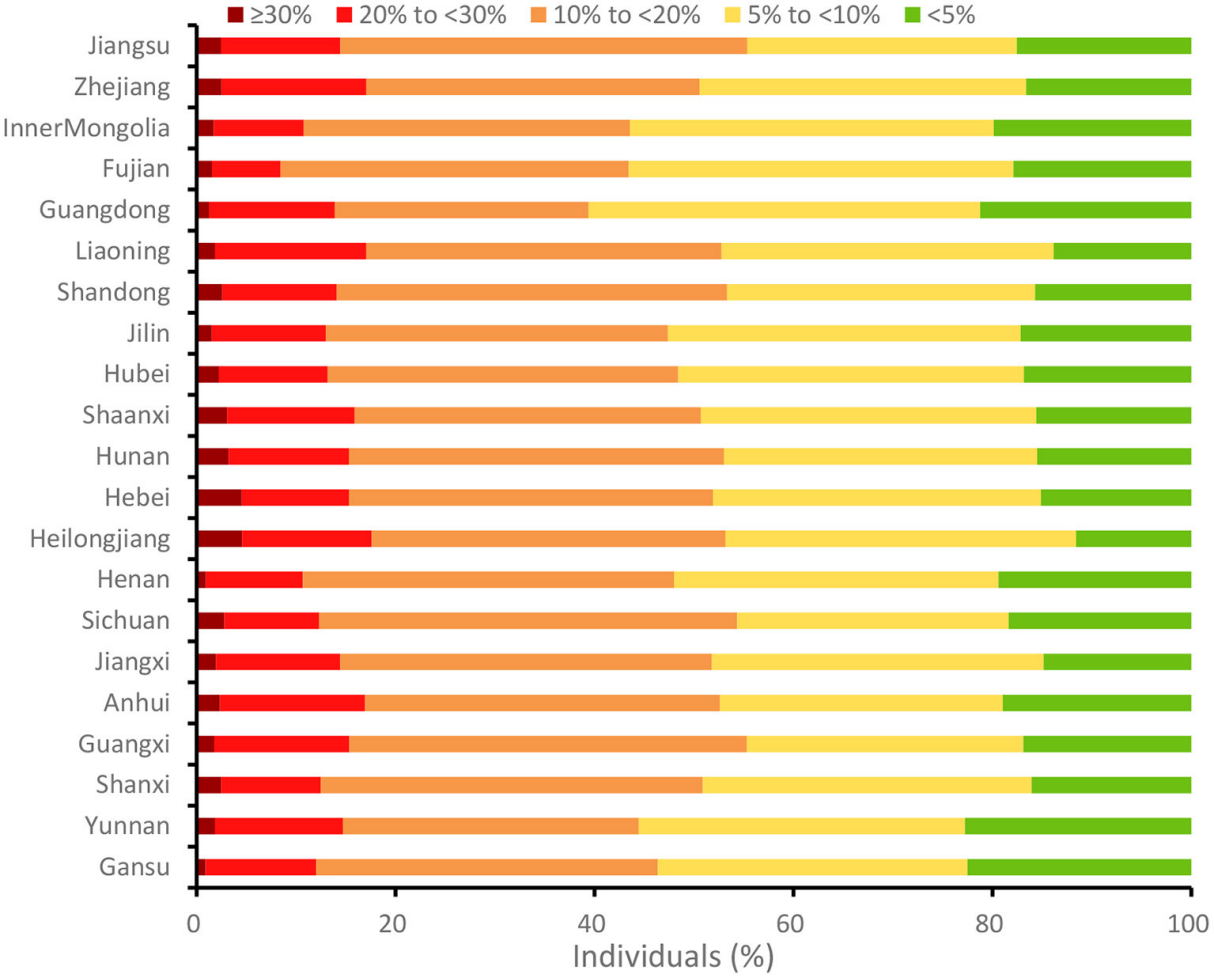
The WHO CVD risk profile for Chinese adults aged 45 years and above by province, 2015. The recently updated non-laboratory-based WHO CVD risk prediction charts were used, which are recommended in the WHO Package of Essential Noncommunicable Disease Interventions. The higher the proportion, the higher the CVD risk.

At the national level, the average of WHO CVD risk score was 11.5, with 14.1% of the 12,597 respondents classified as high-risk (at least a 20% chance of getting CVD; Supplementary Table S3). Figure 1 depicts the 10-year CVD risk distribution for middle-aged and older Chinese individuals by province. For persons aged 45 years and older, the estimated CVD risk varied by province. When we applied recalibrated WHO non-laboratory models to the CHARLS data, we found that the average proportion of adults aged 45 years and older with a risk threshold of 20% was 2.2%, ranging from 0.9% in Gansu to 4.6% in Heilongjiang. Concerning an estimated risk of >10%, the risk factor profile ranged from 39.4% in Guangdong to 55.34% in Guangxi and Jiangsu. In comparison to other provinces in China, Heilongjiang (1.6%) had the lowest level (<5%) and Yunan had the greatest (22.8%).

**Figure 2:**
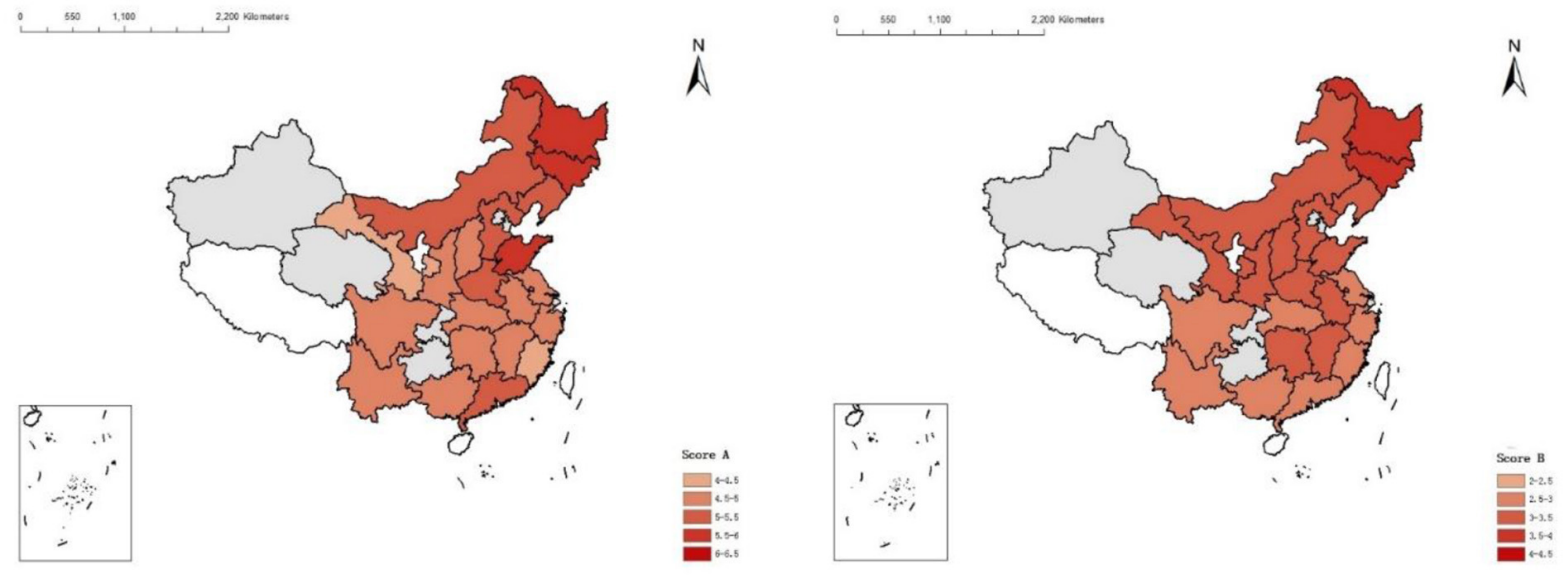
The levels of cardiovascular risk and behavior risk by provinces in China, 2015. The gray area refers to provinces with a small sample size (<200 individuals). The white area refers to provinces without data available in CHARLS. CRS, Cardiovascular Risk Score; BRS, Behavior Risk Score. The higher the score, the higher the CVD risk.

The average CRS and BRS for the full sample of provinces were 4.98 [95% confidence interval (CI) = 4.92, 5.05] and 3.10 (95% CI = 3.04, 3.15), respectively (Supplementary Table S3). The weighted mean CRS (BRS) varied significantly between Fujian and Jilin, ranging from 4.36 (3.71) to 5.72 (2.59). The regional structure of the provincial CVD and BRS is depicted in Figure 2. The CRS was significantly greater in the north-eastern region, comprising Heilongjiang and Jilin, and significantly lower in the south-east region, including Fujian and Zhejiang. Figure 2 reveals similar patterns for the BRS.

### Care cascade for hypertension, diabetes, and dyslipidaemia at national and provincial levels

**Figure 3:**
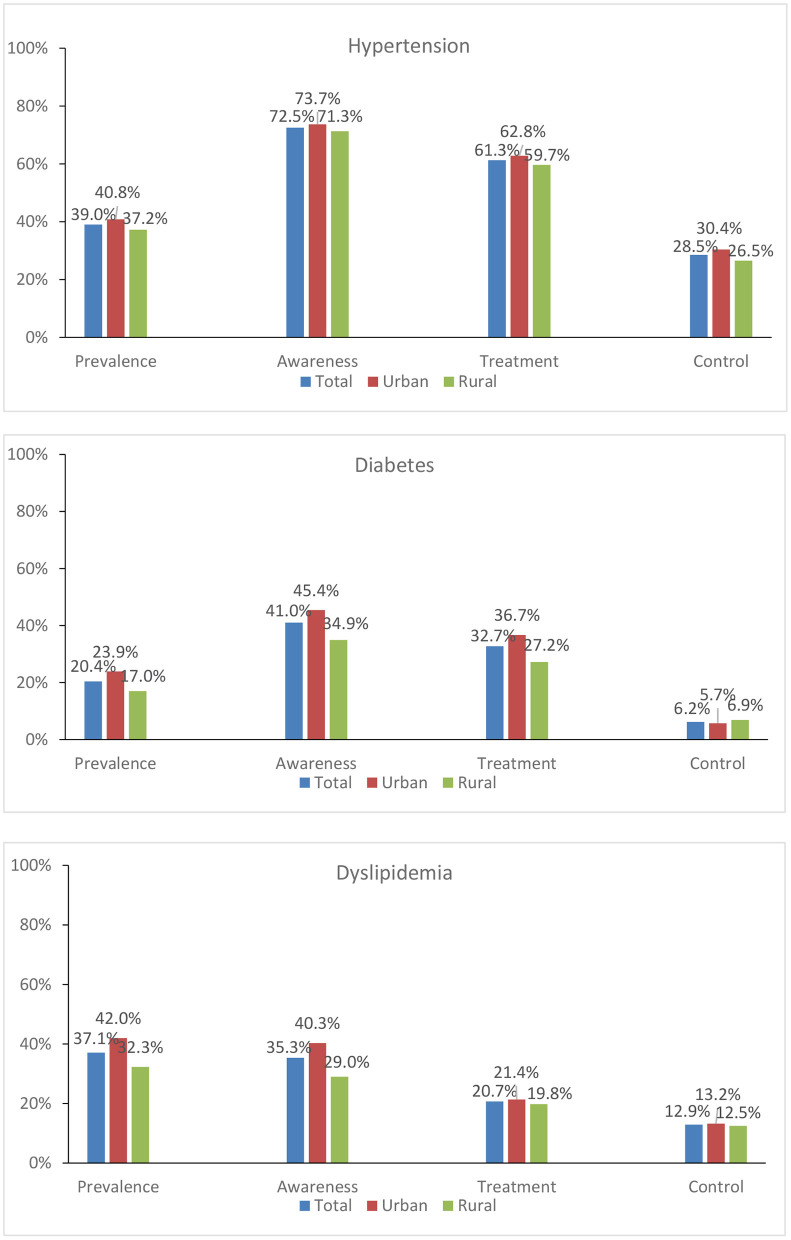
The prevalence and cascade of care for hypertension, diabetes and dyslipidaemia in China.

In 2015, the weighted prevalence of hypertension, diabetes, and dyslipidaemia was 39.0, 20.4, and 37.1%, respectively, among Chinese individuals aged 45 years and older. Indicators of the care cascade for three CVDs examined are shown in Table 1 by sociodemographic category. In total, 72.5% of patients with hypertension were aware of their disease, 61.3% sought treatment, and 28.5% had their blood pressure under control. 41.0% of diabetics were aware of their illness, 32.7% sought treatment, and 6.2% had their diabetes under control. In terms of dyslipidaemia care, 35.3% of those with the condition were aware of it, 20.7% had sought therapy, and 12.9% were “managed.” Rural residents generally had lower results across all care cascade indicators (except for diabetes control) than urban residents (Figure 3).

**Table 1:** CVD prevalence and outcome at national level by socio-demographic groups.

**Variables**	**Prevalence**	**Awareness**	**Treatment**	**Control**
**Hypertension (** ***N*** **)**	12,597	4,951	4,951	4,951
**Overall**	39.0%	72.5%	61.3%	28.5%
Age
45–54	26.0%	65.4%	53.9%	25.2%
55–64	39.1%	73.6%	61.9%	30.7%
65–74	50.3%	77.7%	66.7%	30.1%
75 and above	57.9%	70.8%	60.6%	25.5%
Gender
Male	40.0%	67.2%	54.2%	24.2%
Female	38.0%	77.9%	68.5%	33.0%
Residence place
Urban	40.8%	73.7%	62.8%	30.4%
Rural	37.2%	71.3%	59.7%	26.5%
PCE, quartile
Q1 (the lowest)	39.5%	68.6%	58.2%	23.0%
Q2	40.4%	68.4%	55.6%	25.0%
Q3	37.8%	72.5%	62.5%	32.1%
Q4 (the highest)	38.6%	79.0%	67.4%	32.4%
**Diabetes (** ***N*** **)**	12,597	2,433	2,433	2,433
Overall	20.4%	41.0%	32.7%	6.2%
Age
45–54	13.1%	35.7%	28.4%	6.2%
55–64	23.7%	42.8%	35.0%	5.8%
65–74	23.8%	43.7%	35.0%	6.9%
75 and above	26.5%	39.0%	28.2%	6.0%
Gender
Male	21.0%	36.8%	30.6%	6.2%
Female	19.9%	45.3%	34.9%	6.1%
Residence place
Urban	23.9%	45.4%	36.7%	5.7%
Rural	17.0%	34.9%	27.2%	6.9%
PCE, quartile
Q1 (the lowest)	19.0%	32.3%	25.2%	6.1%
Q2	17.6%	36.3%	28.9%	3.7%
Q3	20.3%	46.0%	36.7%	6.4%
Q4 (the highest)	23.8%	44.9%	36.2%	7.6%
**Dyslipidaemia (** ***N*** **)**	12,597	4,489	4,489	4,489
Overall	37.1%	35.3%	20.7%	12.9%
Age
45–54	33.3%	25.3%	12.8%	8.2%
55–64	41.1%	39.2%	23.4%	13.6%
65–74	39.6%	42.0%	27.1%	18.1%
75 and above	30.2%	35.2%	18.6%	12.3%
Gender
Male	38.5%	34.1%	18.5%	10.9%
Female	35.8%	36.7%	23.0%	15.0%
Residence place
Urban	42.0%	40.3%	21.4%	13.2%
Rural	32.3%	29.0%	19.8%	12.5%
PCE, quartile
Q1 (the lowest)	33.7%	25.6%	16.4%	10.9%
Q2	35.0%	29.3%	16.6%	10.5%
Q3	37.9%	33.7%	22.0%	13.4%
Q4 (the highest)	40.5%	46.9%	25.1%	15.4%

CVD, cardiovascular disease; PCE, per capita household consumption expenditure.

**Figure 4:**
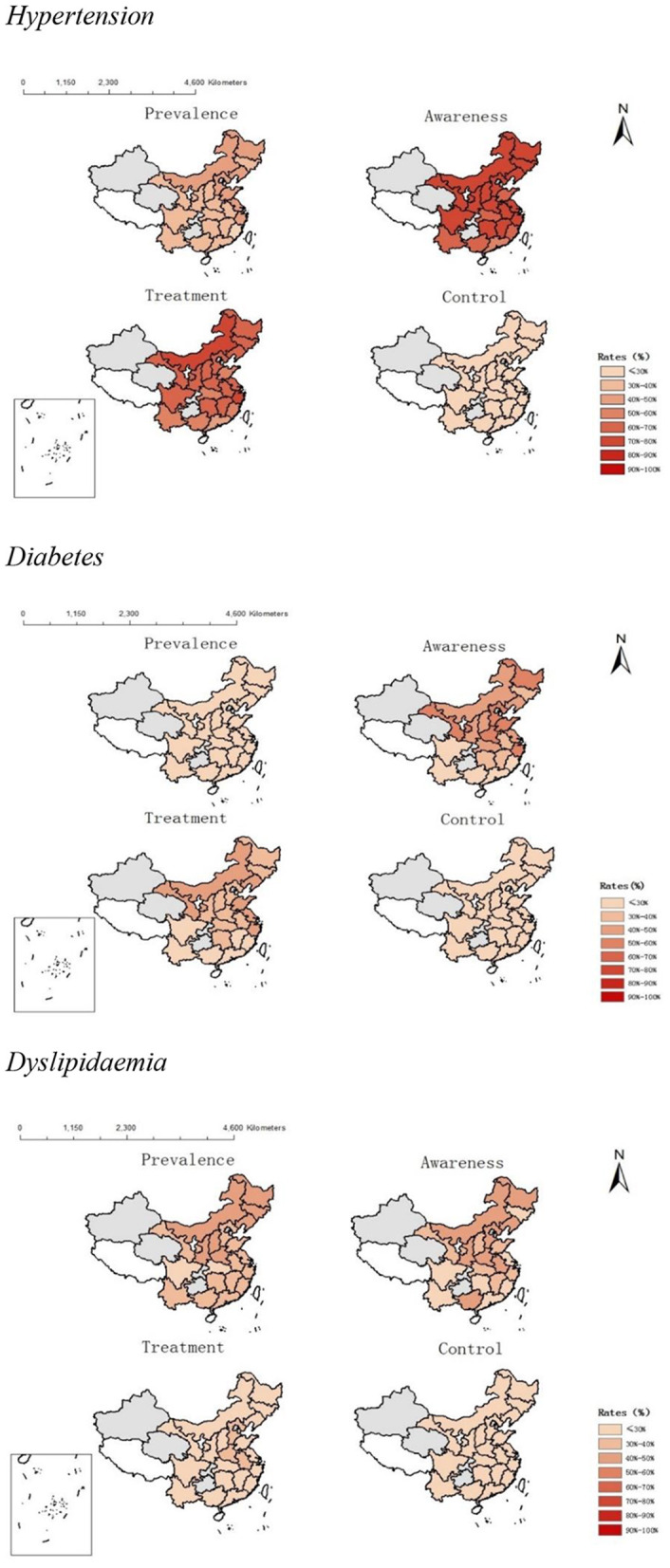
Provincial-level variation in cascade of care for hypertension, diabetes and dyslipidaemia in China. The gray area refers to provinces with a small sample size (<200 individuals). The white area refers to provinces without data available in CHARLS.

**Figure 5:**
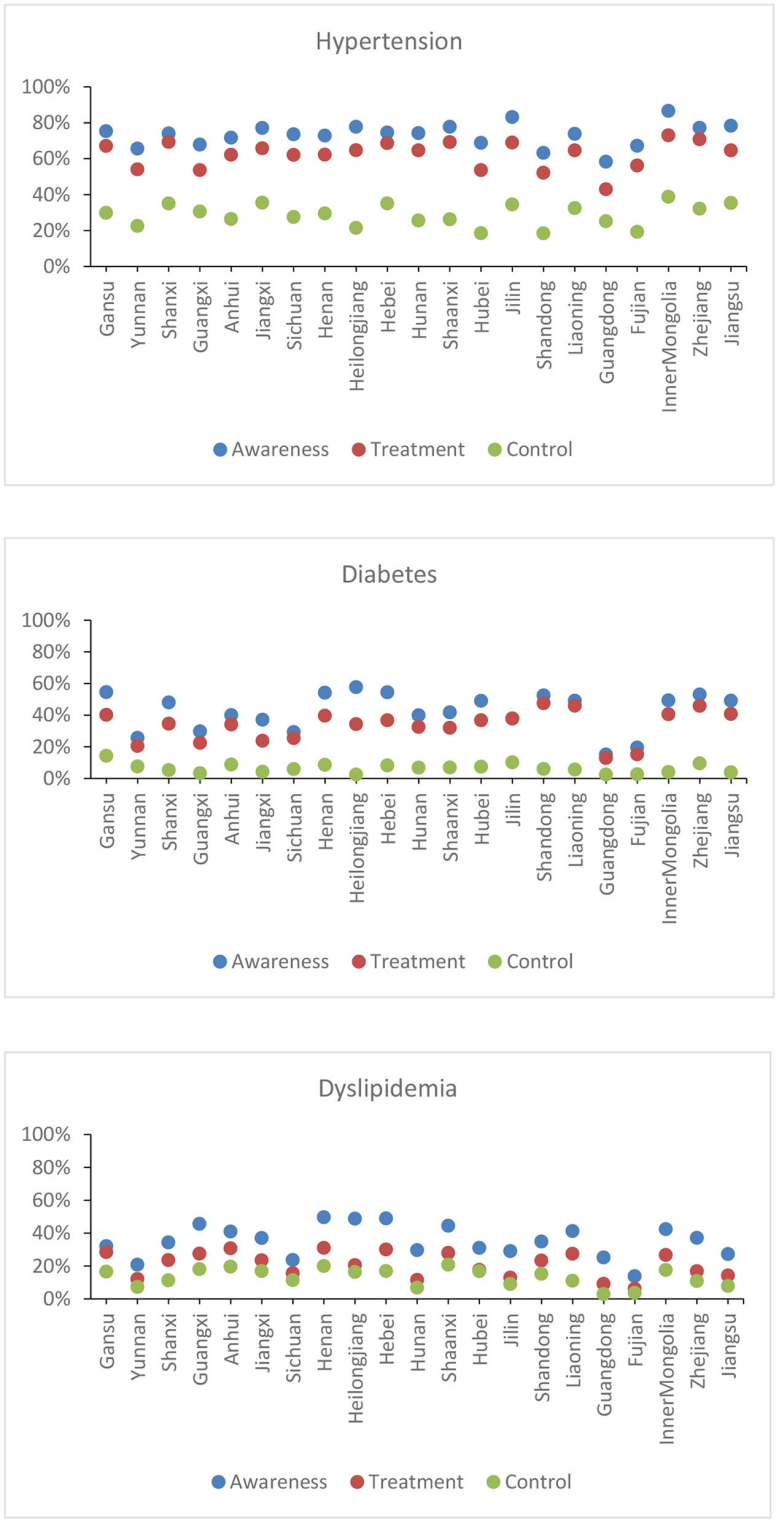
Association between economic development and CVD care cascade indicators. Provinces are ranked according to the economic development in 2015 (The lowest GDP per capita, Gansu; the highest GDP per capita, Jiangsu).

In China, there are disparities in the management of the care cascade for hypertension, diabetes, and dyslipidaemia among provinces (Figure 4). In general, more middle-aged and older adults in China's provinces were aware of and treated for hypertension than for diabetes and dyslipidemia. Inner Mongolia province had the highest prevalence of hypertension awareness (86.6%), treatment (73.0%), and control (38.8%), while Guangdong had the lowest prevalence of hypertension awareness (58.3%) and treatment (43.0%), but Shandong had the highest prevalence of hypertension control (38.8%; 18.4%). Diabetes awareness levels were highest in Northern provinces (Heilongjiang, Gansu, and Hebei) and lowest in southern provinces (Guangdong, Fujian), ranging from 57.7 to 15.1%. Shandong had the greatest treatment rate (47.5%), while Guangdong had the lowest (12.9%). Gansu had the highest control percentage (14.3%), while Guangdong had the lowest (2.4%). Henan province had the highest prevalence of dyslipidaemia awareness (49.6%), treatment (31.0%), and control (19.9%), while Fujian had the lowest prevalence of dyslipidaemia awareness (13.8%) and treatment (6.2%), but Guangdong had the lowest prevalence of dyslipidaemia control (19.9%; 2.9%). There were no discernible correlations between provincial GDP per capita and quality of care for hypertension, diabetes, and dyslipidemia (Figure 5). Provinces with high economic growth have no propensity to have superior quality of care for hypertension, diabetes, and dyslipidaemia.

### Cardiovascular health by individual-level socio-demographic characteristics

Table 2 revealed that the CVD risk was associated with gender (β = −0.349, 95% CI = −0.374, −0.323), high level of education (β = 0.081, 95% CI = 0.007, 0.154), and household' wealth (for the highest level of PCE, β = 0.075, 95% CI = 0.047, 0.103). Those individuals with a higher level of education (β = 0.097, 95% CI = 0.010, 0.185) and wealth status (for the highest level of PCE, β = 0.080, 95% CI = 0.046, 0.114) were also more likely to have high BRS. Similarly, being female had a lower probability of unhealthy behaviors among middle-aged and older adults in China (β = −0.593, 95% CI = −0.626, −0.559).

**Table 2:** Association between cardiovascular risk and socio-demographic characteristics.

**Variables**	**WHO CVD risk score**				**Behavior risk score**			
	**β**	***P*-value**	**95% CI**		**β**	***P*-value**	**95% CI**	
**Education status (illiterate)**
Primary school	0.005	0.743	−0.026	0.037	0.026	0.191	−0.013	0.064
Secondary school	−0.008	0.757	−0.058	0.042	−0.004	0.937	−0.092	0.085
College & above	0.081	0.031	0.007	0.154	0.097	0.029	0.010	0.185
**PCE, quartile (Q1, the lowest)**
Q2	0.029	0.077	−0.003	0.061	0.011	0.671	−0.040	0.063
Q3	0.038	0.023	0.005	0.072	0.038	0.068	−0.003	0.079
Q4 (the highest)	0.075	<0.001	0.047	0.103	0.080	<0.001	0.046	0.114
**Gender (male)**
Female	−0.349	<0.001	−0.374	−0.323	−0.593	<0.001	−0.626	−0.559
**Marital status (married)**
Unmarried	0.021	0.186	−0.010	0.051	0.026	0.199	−0.014	0.065

CVD, cardiovascular disease; PCE, per capita household consumption expenditure.

### Individual correlates of the management of cascade of care for CVDs

Tables 3–5 illustrate covariate-adjusted logistic regressions of hypertension, diabetes, and dyslipidaemia care indicators on individuals' socio-demographic characteristics. Greater education was linked with a higher rate of treated and managed hypertension [Adjusted Odds Ratio (AOR) = 2.46, 95% CI = 1.07, 5.61; OR = 2.32, 95% CI = 1.07, 5.01 separately]. The wealthy group had the highest prevalence of hypertension awareness (AOR = 1.815, 95% CI = 1.381, 2.387), therapy (AOR = 1.536, 95% CI = 1.219, 1.936), and control (AOR = 1.510, 95% CI = 1.203, 1.894). Although males were more aware of their hypertension, females were more likely to treat (AOR = 1.93, 9 CI = 1.594, 2.338) and control their hypertension (AOR = 1.663, 95% CI = 1.355, 2.040). The marital status and hypertension care cascade indicators had a negative connection. Unmarried people were less likely to be aware of their hypertension (AOR = 0.73, 95% CI = 0.57, 0.94), to receive treatment (AOR = 0.71, 95% CI = 0.56, 0.89), and to be under control (AOR = 0.71, 95% CI = 0.55, 0.91).

**Table 3:** Covariate-adjusted logistic regressions of hypertension care cascade indicators on socio-demographic characteristics.

**Variables**	**Awareness**				**Treatment**				**Control**			
	**AOR**	***P*-value**	**95% CI**		**AOR**	***P*-value**	**95% CI**		**AOR**	***P*-value**	**95% CI**	
**Education status (illiterate)**
Primary school	1.068	0.659	0.797	1.433	1.006	0.966	0.778	1.301	1.005	0.973	0.778	1.298
Secondary school	0.842	0.509	0.505	1.403	0.717	0.094	0.486	1.059	0.882	0.516	0.605	1.287
College & above	1.583	0.312	0.650	3.860	2.460	0.032	1.078	5.614	2.323	0.032	1.075	5.017
**PCE, quartile (Q1, the lowest)**
Q2	1.086	0.538	0.836	1.410	0.982	0.883	0.776	1.244	1.149	0.254	0.905	1.459
Q3	1.304	0.048	1.003	1.696	1.289	0.030	1.024	1.623	1.599	<0.001	1.244	2.056
Q4 (the highest)	1.815	<0.001	1.381	2.387	1.536	<0.001	1.219	1.936	1.510	<0.001	1.203	1.894
**Gender (male)**
Female	0.734	<0.001	1.474	2.271	1.931	<0.001	1.594	2.338	1.663	<0.001	1.355	2.040
**Marital status (married)**
Unmarried	0.734	0.015	0.572	0.942	0.712	0.003	0.569	0.892	0.716	0.008	0.558	0.918

AOR, adjusted odds ratio; CI, confidence interval; PCE, per capita household consumption expenditure.

**Table 4:** Covariate-adjusted logistic regressions of diabetes care cascade indicators on socio-demographic characteristics.

**Variables**	**Awareness**				**Treatment**				**Control**			
	**AOR**	***P*-value**	**95% CI**		**AOR**	***P*-value**	**95% CI**		**AOR**	***P*-value**	**95% CI**	
**Education status (illiterate)**
Primary school	1.480	0.012	1.089	2.012	1.275	0.146	0.919	1.769	1.067	0.837	0.576	1.974
Secondary school	1.017	0.953	0.586	1.765	1.009	0.974	0.580	1.756	1.258	0.635	0.488	3.246
College & above	1.283	0.706	0.352	4.676	1.224	0.730	0.388	3.861	0.411	0.257	0.088	1.912
**PCE, quartile (Q1, the lowest)**
Q2	1.202	0.301	0.848	1.704	1.199	0.330	0.832	1.729	0.643	0.141	0.356	1.158
Q3	1.729	0.004	1.187	2.518	1.646	0.013	1.110	2.440	1.203	0.524	0.681	2.125
Q4 (the highest)	1.559	0.033	1.037	2.345	1.518	0.049	1.001	2.302	1.531	0.164	0.840	2.793
**Gender (male)**
Female	1.497	0.006	1.122	1.997	1.275	0.109	0.947	1.715	0.938	0.805	0.563	1.561
**Marital status (married)**
Unmarried	0.979	0.901	0.705	1.361	0.910	0.595	0.644	1.287	1.640	0.095	0.918	2.928

AOR, adjusted odds ratio; CI, confidence interval; PCE, per capita household consumption expenditure.

**Table 5:** Covariate-adjusted logistic regressions of dyslipidaemia care cascade indicators on socio-demographic characteristics.

**Variables**	**Awareness**				**Treatment**				**Control**			
	**AOR**	***P*-value**	**95% CI**		**AOR**	***P*-value**	**95% CI**		**AOR**	***P*-value**	**95% CI**	
**Education status (illiterate)**
Primary school	1.702	<0.001	1.326	2.183	1.107	0.504	0.821	1.494	1.013	0.942	0.713	1.439
Secondary school	1.657	0.011	1.121	2.448	1.306	0.184	0.881	1.937	1.484	0.079	0.955	2.308
College & above	4.185	<0.001	2.134	8.207	0.959	0.899	0.508	1.813	1.079	0.838	0.519	2.243
**PCE, quartile (Q1, the lowest)**
Q2	1.218	0.206	0.897	1.654	1.088	0.563	0.817	1.449	1.038	0.819	0.754	1.429
Q3	1.424	0.029	1.037	1.956	1.572	0.003	1.167	2.118	1.393	0.066	0.978	1.982
Q4 (the highest)	2.102	<0.001	1.514	2.918	1.771	<0.001	1.297	2.419	1.523	0.014	1.091	2.127
**Gender (male)**
Female	1.301	0.012	1.060	1.596	1.376	0.007	1.092	1.732	1.496	0.004	1.137	1.969
**Marital status (married)**
Unmarried	0.844	0.204	0.649	1.097	0.865	0.334	0.645	1.161	0.833	0.316	0.584	1.190

AOR, adjusted odds ratio; CI, confidence interval; PCE, per capita household consumption expenditure.

Covariate-adjusted logistic regressions of diabetes care cascade indicators on socio-demographic factors (Table 4) revealed that participants who had completed elementary school were more likely to have a high level of diabetes awareness (AOR = 1.48, 95% CI = 1.08, 2.012). Diabetes awareness and treatment level were connected with higher levels of household consumption expenditure. Diabetes awareness (for those in Q3, AOR = 1.729, 95% CI = 1.18, 2.51) and treatment (for those in Q3, AOR = 1.64, 95% CI = 1.11, 2.44) were higher among those with higher consumption expenditure. Women, on the other hand, reported being more aware of their diabetes status (AOR = 1.49, 95% CI = 1.12, 1.99) than men. According to Table 5, the prevalence of dyslipidaemia awareness was substantially related to education level. Higher education level was associated with greater dyslipidaemia awareness (AOR = 4.185, 95% CI = 2.134, 8.207). Similarly, females and participants in the top quartile of household expenditure were found to have better management of cascade of care for dyslipidaemia among middle-aged and older Chinese adults.

## Discussion

### Principal findings

Our study revealed that in China the management of cascade of care for diabetes and dyslipidaemia management is poor, with over 67 and 79% of the population, respectively, having untreated diabetes and dyslipidaemia. Additionally, we found a higher prevalence of hypertension awareness and treatment in northern, more rural provinces and a lower prevalence in southern provinces. Diabetes and dyslipidemia followed similar patterns.

Northern provinces (Inner Mongolia, Jilin) performed best in managing cascade of care for hypertension, while Shandong, Hubei province performed worst. For diabetes management, Heilongjiang, Zhejiang province outperformed Guangdong, Fujian province. This was in contrast to management of cascade of care for dyslipidaemia, with Henan and Hebei provinces performing best and Fujian and Guangdong provinces performing worst. CVD risk was higher in the north-eastern region (Heilongjiang and Jilin) and lower in the south-east, when age and gender were standardized (Fujian and Zhejiang). This study revealed similar patterns for BRSs across provinces in China.

Individual-level wealth was related to higher CVD risks, but with improved NCD diagnosis, treatment, and control for hypertension, diabetes, and dyslipidaemia after controlling for other potential confounding variables. Importantly, our study found there is no clear correlation between a province's economic development and the management of cascade of care for hypertension, diabetes, and dyslipidaemia.

### Comparisons of the literature

Our findings regarding regional differences in the prevalence (18, 19) and overall levels of care cascade indicators for are consistent with previous reports (20). The prevalence of CVDs and CVD risks are consistent north-south across studies in China (21–23). According to a systematic review of 47 studies published in China between 2002 and 2012, the prevalence of age-standardized hypertension was higher in northern provinces (30.4% in the middle north, 28.3% in the north-east, and 23.6% in the north-west) and lower in eastern and southern provinces (23.2% in the east, 16.2% in the middle south, and 19.9% in the southwest) (24, 34).

Provincial differences in hypertension awareness, treatment, and control were significant in other studies, although the gap between the indicators varied (35). The differences in indicator definitions and data collection methods (self-reported or validated measures) across studies could account for the majority of the gap range discrepancies (36). Several studies examined geographic variations by grouping all participants into six broad regions (East China, North China, Northeast China, Northwest China, South Central China, and Southwest China), rather than by province (18), which may result in discrepancies in measuring regional economic development. Variation in self-reported or validated measures of CVDs may also account for the discrepancy with previous studies (36). Tang and colleagues discovered that provinces with a medium GDP per capita have the lowest prevalence of hypertension when compared to those with a high or low GDP per capita (37). Compared to previous studies, this current study has a higher degree of homogeneity in the demographics of middle-aged and older participants, which may result in insufficient variation to detect a significant association.

We found that individuals with a higher level of education and wealth status were more likely to have high CVD risk and chronic conditions. Our findings contrast with evidence from high-income countries (38, 39). For instance, a study of 16 European countries, found that individuals in the highest income quintile and those with higher levels of education were less likely to have multimorbidity than those with low levels of income and education (38). This may be due to LMICs being in an earlier stage of the epidemiological transition compared to high-income countries (28, 29). Our findings are consistent with other studies in LMICs (e.g., India and Bangladesh) and found that obesity, physical inactivity, and consumption of tobacco, alcohol, fat, salt, and processed food are more prevalent among higher socio-economic groups (40, 41).

People in higher-income and education level groups, who have better access to healthcare services and better health literacy, are more likely to have NCDs diagnosed (or even over-diagnosed) than lower socio-income groups (40, 42). Further, the recall of specific chronic conditions and risk factors that have been diagnosed some time ago, may be higher in those of higher socio-economic groups (for example, because they are paying for ongoing treatment). The smaller capacity and lower accessibility of rural facilities could mean that CVD risk is under-reported in rural areas. Our results may reflect a combination of true underlying prevalence, access to health care, and health literacy.

### Policy implications

Our findings indicate that there are no clear associations between provincial economic development and CVDs management. Socioeconomic disparities in NCD management are severe in both high-development provinces (such as Fujian) and low-development provinces (such as Gansu province). These findings imply that economic development by itself does not result in reductions in socioeconomic inequalities in NCD management. This implies that the higher levels of expenditure of more prosperous provinces are not applied to addressing the barriers to quality care that Chinese individuals with low socio-economic status face. These are already well understood to include provisions within the Chinese health insurance system including monthly reimbursement caps, co-payments that impose a significant financial burden on poorer households and discrepancies in coverage levels between Provinces and between rural and urban areas. The implications of these provision are particularly marked for those suffering from NCDs which are predominantly chronic and therefore impose their financial burdens chronically, levying co-payments and deductibles month after month with more insidious and poverty trapping impacts than for acute illnesses with once-off financial implications.

The prevalence and incidence of CVDs are increasing in China, and substantial geographical and rural–urban variations in prevalence and healthcare exist, which will re-emphasize the importance of implementing policy strategies for the prevention and management of these serious diseases. Population-wide programs and healthcare systems should increase efforts on prevention, and in facilitating access to proven interventions and quality of care for disadvantaged segments of the population (43, 44). A focus on the accessibility to strategies for the control of risk factors in rural areas is especially pertinent, where there is a substantially greater burden of CVDs. In China, people's awareness of healthy lifestyles and behavior is still suboptimal (44). Further action to educate the public on health, screening those at high risk, and population-wide management of CVD risk factors are required.

Provincial governments should use growing availability of resources for the health system to prioritize reducing the financial burdens of ill health experienced by the poorest households through increasing the generosity of the provisions of their insurance systems, targeting categories of insured according to their socio-economic disadvantage. This will be an important economic development strategy as well as an investment in equity and social welfare. The impact of the failure to address NCDs in poorer households is important for productivity through the remainder of the life cycle, early death and impoverishment through out-of-pocket spending, all of which influence the life chances of all members of the household, economic activity levels and the onward inter-generational transmission of poverty.

Significant global and national initiatives, such as the Healthy China 2030 vision and the World Health Organization's Global NCD Targets, have attempted to address the issue of increasing exposure to risk factors and the growing burden of NCDs (45, 46). Differences in CVD and behavioral risk scores and coverage levels across NCD care cascades, suggest the need for geographically and population-based targeted approaches. Provinces should review their specific profiles across the care cascades to understand where greater investment is needed in terms of service provision and health education initiatives. Our results indicate that there is no one-size-fits-all approach at the national level.

Nevertheless, it is likely that all Provinces will benefit from prioritizing investments in primary care over those in hospital care for both economic and social welfare impact. At all three stages of the care cascade, awareness, accessing treatment and achieving control, there are effective and cost-effective opportunities at the primary care level. Costs for both insurance system and service users are lower at primary care level and therefore financial barriers more capable of being addressed. Adaptations to increase coverage completeness targeted at outpatient and primary care coupled with investments in service availability and quality in primary care facilities are likely to be the vehicles for rapid and effective closing of the gaps that this study has identified.

### Strengths and weaknesses

The primary strength of this study is the use of a nationally representative dataset to collect comprehensive biomedical data that allows for more reliable estimates of hypertension, diabetes, and dyslipidemia prevalence (47). Additionally, measuring CVD risk and behavioral risk scores at the provincial level provides compelling evidence about local epidemiology and the disproportionate burden of NCD across geographic areas. This information is critical for refining current strategies for preventing premature death from NCD. However, these findings should be interpreted within the context of some limitations. First, the study's cross-sectional design precluded establishing causal relationships between individuals' sociodemographic characteristics and NCD risk factors and management. Our findings warrant further investigation by examining the trend in risk factors over time using time series or longitudinal data. Second, because data on behavioral risk factors such as tobacco use, alcohol consumption, and physical activity were self-reported, recall bias as well as social acceptability bias may have influenced the risk scores. Additionally, the tobacco and alcohol use definitions used to generate the BRS did not take quantity and frequency of consumption into account. Third, there might be the *de novo* diagnoses of hypertension, diabetes and dyslipidaemia, which this cross-sectional study could not identify. Finally, the CHARLS only included middle-aged and older adults in China. The prevalence of hypertension, diabetes, and dyslipidemia as well as CVD risk among younger populations should be considered in future studies.

## Data availability statement

The original contributions presented in the study are included in the article/Supplementary material, further inquiries can be directed to the corresponding author.

## Ethics statement

The Biomedical Ethics Review Committee of Peking University approved the CHARLS study (approval number: IRB00001052–11015), and all interviewees were required to provide informed consent. The patients/participants provided their written informed consent to participate in this study.

## Author contributions

YZ and JL conceived and designed the study. YZ and KA carried out the initial analysis. RA, and BM interpreted the data. YZ and ND analyzed the literature. YZ, KA, and JL wrote the first draft of the paper. RA, TM, CH, BM, ND, EH, XF, YD, BO, and JL provided advice on the first draft and revised the article critically for important intellectual content. All authors reviewed and had final approval of the submitted and published versions.

## Conflict of interest

The authors declare that the research was conducted in the absence of any commercial or financial relationships that could be construed as a potential conflict of interest.

## Publisher's note

All claims expressed in this article are solely those of the authors and do not necessarily represent those of their affiliated organizations, or those of the publisher, the editors and the reviewers. Any product that may be evaluated in this article, or claim that may be made by its manufacturer, is not guaranteed or endorsed by the publisher.
