# Supplementary material for: Provincial heterogeneity in the management of care cascade for hypertension, diabetes, and dyslipidaemia in China: Analysis of nationally representative population-based survey
Source: Front Cardiovasc Med. 2022 Aug 23;9:923249. doi: 10.3389/fcvm.2022.923249 (PMC9458474; doi:10.3389/fcvm.2022.923249)
Supplement: Supplementary file 1 [file Data_Sheet_1.docx]

**Appendix**

**Fig S1**. Flowchart showing the selection of the subjects who were included in the final analysis, 2015

Exclude due to: Missing value of socio-demographic data (n=416)

Exclude due to: Missing value of the weighting variable (n=341)

All individuals in CHARLS 2015 (n=20,197)

Exclude due to: Age younger than 45 years old (n=1,258)

18,939 individuals left

Exclude due to: Missing biomarkers and/or blood test (n=5,585)

12,597 individuals included in final analysis

13,354 individuals left

13,013 individuals left

**Table S1.** Characteristics of participants

| **Characteristic** | **N** | **% (1)** | **% (2)** |
| --- | --- | --- | --- |
| Total | 12,597 | 100 | 100 |
| Age (year) |  |  |  |
| 45-54 | 3,949 | 31.4 | 33.5 |
| 55-64 | 4,471 | 35.5 | 34.7 |
| 65-74 | 3,046 | 24.2 | 22.0 |
| 75 and above | 1,131 | 9.0 | 9.7 |
| Gender |  |  |  |
| Male | 5,929 | 47.1 | 49.2 |
| Female | 6,668 | 52.9 | 50.8 |
| Marital status |  |  |  |
| Married and partnered | 10,908 | 86.6 | 86.5 |
| Unmarried and other | 1,689 | 13.4 | 13.5 |
| Education status |  |  |  |
| Illiterate | 8,920 | 70.8 | 65.9 |
| Primary school | 2,453 | 19.5 | 20.3 |
| Secondary school | 1,062 | 8.4 | 11.0 |
| College & above | 162 | 1.3 | 2.8 |
| PCE, quartile |  |  |  |
| Q1, the lowest | 3,151 | 25.0 | 20.5 |
| Q2 | 3,148 | 25.0 | 23.8 |
| Q3 | 3,150 | 25.0 | 27.0 |
| Q4 (the highest) | 3,148 | 25.0 | 28.7 |
| Residence place |  |  |  |
| Urban | 4,762 | 37.8 | 49.6 |
| Rural | 7,835 | 62.2 | 50.4 |

Note: % (2), the values are weighted percentages unless otherwise indicated.

PCE, Per capita household consumption expenditure.

**Table S2.** Definitions of poor, intermediate, and ideal levels of seven cardiovascular health metrics in Chinese adults aged ≥45 years

| **Metrics** | **Poor health** | **Intermediate health** | **Ideal health** |
| --- | --- | --- | --- |
| Current smoking | Yes | Former, quit ≤12 months | Never or quit >12 months |
| Current drinking | Yes | Former, quit ≤12 months | Never or quit >12 months |
| Physical activity | Those individuals who not meet criteria for categories ‘intermediate health’ or ‘ideal health ‘for physical activity | a) 3 or more days of vigorous-intensity activity of at least 10 minutes per day but less than 30 minutes; b) 5 or more days of moderate-intensity activity and/or walking of at least 30 minutes per day | 3 or more days of vigorous-intensity activity of at least 30 minutes per day |
| Sleep met guideline | No | - | Yes |
| Body mass index | ≥30.0 kg/m2 | 25.0-29.9 kg/m2 | <25.0 kg/m2 |
| Total cholesterol | ≥240 mg/dl | 200-239 mg/dl or treated to goal | <200 mg/dl |
| Blood pressure | Systolic ≥140 or diastolic ≥90 mm Hg | Systolic 120-139 or diastolic 80-89 mm Hg or treated to goal | <120/<80 mm Hg |
| Fasting plasma glucose | ≥126 mg/dl | 100-125 mg/dl or treated to goal | <100 mg/dl |

Poor sleep was defined as those who do not meet recommended sleep duration, either less than 7 hours per day or more than 9 hours.

**Table S3.** The CVD risk and behaviour risk by provinces in China, 2015.

| **Province** | **N** | **Score A (CRS)** | **Score B (BRS)** | **Proportion of**  **WHO CVD risk level** | | | | |
| --- | --- | --- | --- | --- | --- | --- | --- | --- |
|  |  |  |  | <5% | 5%-<10% | 10%-<20% | 20%-<30% | ≥30% |
| **Overall** | 12, 022 | 4.98 | 3.10 | 17.5% | 32.5% | 35.8% | 12.0% | 2.2% |
| Jiangsu | 611 | 4.85 | 2.99 | 17.5% | 27.1% | 40.9% | 12.0% | 2.4% |
| Zhejiang | 553 | 4.84 | 2.94 | 16.6% | 32.9% | 33.5% | 14.5% | 2.5% |
| InnerMongolia | 562 | 5.31 | 3.39 | 19.9% | 36.6% | 32.8% | 9.0% | 1.7% |
| Fujian | 333 | 4.36 | 2.59 | 17.9% | 38.7% | 35.0% | 6.9% | 1.5% |
| Guangdong | 454 | 5.03 | 2.94 | 21.2% | 39.4% | 25.5% | 12.6% | 1.2% |
| Liaoning | 395 | 5.27 | 3.28 | 13.8% | 33.4% | 35.7% | 15.2% | 1.8% |
| Shandong | 1,287 | 5.59 | 3.41 | 15.7% | 31.0% | 39.2% | 11.5% | 2.6% |
| Jilin | 283 | 5.72 | 3.71 | 17.2% | 35.5% | 34.4% | 11.5% | 1.5% |
| Hubei | 313 | 4.74 | 2.95 | 16.8% | 34.8% | 35.2% | 11.0% | 2.2% |
| Shaanxi | 471 | 4.85 | 3.03 | 15.6% | 33.7% | 34.8% | 12.8% | 3.0% |
| Hunan | 533 | 4.99 | 3.04 | 15.5% | 31.5% | 37.7% | 12.1% | 3.2% |
| Hebei | 580 | 5.13 | 3.28 | 15.1% | 33.0% | 36.6% | 10.8% | 4.5% |
| Heilongjiang | 234 | 5.68 | 3.53 | 11.6% | 35.2% | 35.6% | 13.0% | 4.6% |
| Henan | 1,075 | 5.04 | 3.16 | 19.4% | 32.6% | 37.3% | 9.8% | 0.9% |
| Sichuan | 991 | 4.76 | 2.95 | 18.4% | 27.3% | 42.0% | 9.5% | 2.8% |
| Jiangxi | 676 | 4.68 | 3.08 | 14.9% | 33.3% | 37.4% | 12.5% | 1.9% |
| Anhui | 748 | 4.99 | 3.21 | 19.0% | 28.5% | 35.7% | 14.6% | 2.3% |
| Guangxi | 410 | 4.78 | 2.78 | 16.9% | 27.8% | 40.0% | 13.6% | 1.8% |
| Shanxi | 393 | 4.83 | 3.24 | 16.1% | 33.1% | 38.4% | 10.0% | 2.4% |
| Yunnan | 802 | 4.70 | 2.87 | 22.8% | 32.8% | 29.7% | 12.9% | 1.8% |
| Gansu | 318 | 4.46 | 3.04 | 22.5% | 31.1% | 34.3% | 11.1% | 0.9% |

Note: CRS, Cardiovascular Risk Score. BRS, Behaviour Risk Score. WHO CVD risk score, according to recalibrated non-laboratory-based WHO CVD risk charts for Chinese adults.

**Table S4**. The cascade of care for hypertension, diabetes and dyslipidaemia by provinces in China, 2015

| **Province** | **Hypertension** | | | | **Diabetes** | | | | **Dyslipidemia** | | | |
| --- | --- | --- | --- | --- | --- | --- | --- | --- | --- | --- | --- | --- |
|  | Prevalence | Awareness | Treatment | Control | Prevalence | Awareness | Treatment | Control | Prevalence | Awareness | Treatment | Control |
| **Jiangsu** | 41.4% | 78.3% | 64.6% | 35.3% | 23.0% | 49.2% | 40.8% | 3.9% | 38.2% | 27.24% | 14.24% | 7.84% |
| **Zhejiang** | 42.8% | 77.1% | 70.7% | 32.1% | 17.2% | 53.1% | 45.9% | 9.5% | 39.9% | 37.12% | 16.81% | 10.77% |
| **InnerMongolia** | 42.6% | 86.6% | 73.0% | 38.8% | 22.4% | 49.3% | 40.6% | 4.1% | 45.7% | 42.39% | 26.63% | 17.53% |
| **Fujian** | 26.6% | 67.2% | 56.1% | 19.2% | 16.1% | 19.5% | 15.2% | 2.6% | 36.1% | 13.77% | 6.17% | 3.58% |
| **Guangdong** | 37.5% | 58.3% | 43.0% | 25.1% | 25.9% | 15.1% | 12.9% | 2.4% | 39.6% | 25.15% | 9.08% | 2.93% |
| **Liaoning** | 45.2% | 73.7% | 64.6% | 32.5% | 19.9% | 49.1% | 46.0% | 5.6% | 44.8% | 41.17% | 27.38% | 10.95% |
| **Shandong** | 44.9% | 63.2% | 52.1% | 18.4% | 20.6% | 52.5% | 47.5% | 6.0% | 37.7% | 34.82% | 23.28% | 15.15% |
| **Jilin** | 35.8% | 83.1% | 68.9% | 34.5% | 28.3% | 37.9% | 37.9% | 10.2% | 45.1% | 28.99% | 12.85% | 8.95% |
| **Hubei** | 34.2% | 68.8% | 53.6% | 18.5% | 21.9% | 49.1% | 36.8% | 7.4% | 28.3% | 30.95% | 17.59% | 16.77% |
| **Shaanxi** | 39.3% | 77.7% | 69.2% | 26.2% | 18.8% | 41.8% | 32.0% | 6.9% | 40.2% | 44.40% | 27.95% | 20.73% |
| **Hunan** | 38.1% | 74.2% | 64.6% | 25.5% | 22.1% | 39.9% | 32.6% | 6.9% | 36.3% | 29.66% | 11.53% | 6.72% |
| **Hebei** | 49.8% | 74.5% | 68.6% | 35.1% | 21.6% | 54.5% | 36.8% | 8.2% | 32.9% | 48.87% | 30.00% | 16.90% |
| **Heilongjiang** | 47.2% | 77.8% | 64.6% | 21.5% | 22.2% | 57.7% | 34.3% | 2.4% | 41.7% | 48.73% | 20.49% | 16.35% |
| **Henan** | 38.1% | 72.8% | 62.2% | 29.5% | 21.5% | 54.2% | 39.6% | 8.6% | 42.8% | 49.60% | 31.02% | 19.93% |
| **Sichuan** | 37.3% | 73.5% | 62.0% | 27.5% | 19.0% | 29.3% | 25.5% | 5.9% | 26.7% | 23.60% | 15.56% | 11.33% |
| **Jiangxi** | 32.3% | 77.1% | 65.7% | 35.5% | 20.4% | 37.1% | 23.8% | 4.2% | 30.0% | 37.02% | 23.41% | 16.87% |
| **Anhui** | 38.8% | 71.6% | 62.1% | 26.4% | 17.1% | 40.0% | 34.1% | 8.8% | 34.8% | 40.93% | 30.68% | 19.62% |
| **Guangxi** | 32.9% | 67.8% | 53.6% | 30.5% | 18.0% | 29.7% | 22.5% | 3.3% | 31.8% | 45.60% | 27.46% | 18.09% |
| **Shanxi** | 39.2% | 74.1% | 69.2% | 35.0% | 18.2% | 48.0% | 34.6% | 5.3% | 41.2% | 34.27% | 23.49% | 11.27% |
| **Yunnan** | 35.4% | 65.6% | 54.0% | 22.5% | 14.6% | 25.6% | 20.5% | 7.6% | 36.1% | 20.73% | 12.03% | 7.20% |
| **Gansu** | 30.7% | 75.3% | 67.1% | 29.8% | 15.4% | 54.6% | 40.2% | 14.3% | 35.4% | 32.02% | 28.39% | 16.56% |

**Table S5.** Description of the care cascade indicators of hypertension, diabetes, and dyslipidaemia

| **Care cascade indicators** | **Description** |
| --- | --- |
| **Hypertension** |  |
| Prevalence/diagnosis | Percent of population with high blood pressure measured and who were previously diagnosed by a health care professional according to guidelines |
| Awareness | Percent of hypertensive population who had a self-report of any prior diagnosis of hypertension |
| Treatment | Percent of hypertensive population who used pharmacological treatment of hypertension at the time of the interview |
| Control | Percent of hypertensive population with controlled blood pressure |
| **Diabetes** |  |
| Prevalence/diagnosis | Percent of population with diabetes measured and who were previously diagnosed by a health care professional according to standards in guidelines |
| Awareness | Percent of population with high blood sugar who had a self-report of any prior diagnosis of diabetes |
| Treatment | Percent of population with high blood sugar who used pharmacological treatment of diabetes at the time of the interview |
| Control | Percent of diabetes population with controlled blood sugar |
| **Dyslipidaemia** |  |
| Prevalence/diagnosis | Percent of population with high blood lipids measured and who were previously diagnosed by a health care professional according to standards in guidelines |
| Awareness | Percent of population with high blood lipids who had a self-report of any prior diagnosis of dyslipidaemia |
| Treatment | Percent of population with high blood lipids who used pharmacological treatment of dyslipidaemia at the time of the interview |
| Control | Percent of dyslipidaemia population with controlled blood lipids |
